# Haptic Glove Using Tendon-Driven Soft Robotic Mechanism

**DOI:** 10.3389/fbioe.2020.541105

**Published:** 2020-10-08

**Authors:** Siyeon Baik, Shinsuk Park, Jaeyoung Park

**Affiliations:** ^1^Robotics and Media Institute, Korea Institute of Science and Technology, Seoul, South Korea; ^2^Department of Mechanical Engineering, Korea University, Seoul, South Korea

**Keywords:** haptic interface, tendon-driven mechanism, wearable interface, cutaneous feedback, kinesthetic feedback

## Abstract

Recent advancements in virtual reality and augmented reality call for light-weight and compliant haptic interfaces to maximize the task-performance interactivity with the virtual or extended environment. Noting this, we propose a haptic glove using a tendon-driven compliant robotic mechanism. Our proposed interface can provide haptic feedback to two fingers of a user, an index finger and a thumb. It can provide both cutaneous and kinesthetic feedback to the fingers by using the tendon-driven system. Each actuator is paired with a force sensor to exert the desired tension accurately. In order to optimize the perception of the kinesthetic feedback, we propose a perception-based kinesthetic feedback distribution strategy. We experimentally measured the force perception weight for peripheral interphalangeal (PIP) and metacarpophalangeal (MCP) joints. We observed no significant difference in the force perception between the two joints. Then, based on the obtained weights, our proposed force distribution method calculates the force for each joint. We also evaluated the effect of additional cutaneous feedback to the kinesthetic feedback, on the force perception at the fingertip. The experimental result has shown that additional cutaneous feedback has significantly increased the sensitivity of the human perception. Finally, we evaluated our proposed system and force distribution algorithm by conducting a human subject test. The experimental result indicates that the availability of the cutaneous feedback significantly improved the perceived realism and acuity of the contact force.

## 1. Introduction

Haptic interfaces are widely used due to their verified effect in enhancing the presence and the task-performance in the virtual reality (VR) or augmented reality (AR) (Rosenberg, 1995; Sallnäs et al., 2000; Hecht et al., 2006). Generally, haptic interfaces can be categorized depending on their grounding locations (Prattichizzo et al., 2019): grounded devices (Massie and Salisbury, 1994), non-grounded devices (Choi et al., 2018). In early days, various types of grounded haptic interfaces were developed and still dominantly utilized in robotic fields owing to its accurate force application and sufficient stiffness range. The grounded devices, such as stylus/trackball type kinesthetic feedback interfaces, can only deal with fixed workspace due to its limitation in freedom of motion. Meanwhile, non-grounded devices can provide better flexibility in operating the virtual objects. Accordingly the weight of the system is regarded as a crucial factor in order to minimize the users' fatigue. For this reason, tendon-driven haptic interface has been well used since it has light-weighted end-effectors as well as compliant mechanism. The popular form of the tendon-driven glove interface is rendering kinesthetic feedback on a finger by exerting resistive force on its joint. However, few of the studies have evaluated the influence of adequately distributed force-feedback on multiple joints. Besides, previous studies have overlooked the effect of the combined form of kinesthetic feedback and cutaneous feedback. For the improvement, we reckon two factors can make progress of haptic interface: combined feedback of cutaneous and kinesthetic feedback, and the optimization of force distribution on finger joints. Hence, in the present study, we suggest tendon-driven cutaneous and kinesthetic feedback interface with optimized control.

Until now, various types of wearable hand haptic interfaces have been proposed. One example is a cutaneous fingertip interface which stimulates the users' fingertip (e.g., Prattichizzo et al., 2013; Tsetserukou et al., 2014; Gabardi et al., 2016). The cutaneous fingertip interface raises a sensation that users themselves are indeed touching the objects by stimulating mechanoreceptors in virtual environment. Therefore, it can provide spatial cues of virtual objects by giving the information of the orientation and the variations of the contact area. Another form of the haptic feedback interface is a glove type or exoskeleton kinesthetic feedback interface, which operates in the manner of imparting a resistive force on the finger joints(e.g., Bouzit et al., 2002; Blake and Gurocak, 2009; Polygerinos et al., 2015). By letting the users directly feel the contact at their fingers, it emulates the sensation of touching a real object while minimizing the constraint on hand movement. The earlier studies indicate that the majority of haptic engineers have focused on kinesthetic feedback rather than on cutaneous feedback when designing the haptic gloves. However, to maximize the sense of reality in VR, haptic displays with the force and the tactile feedback are essential (Srinivasan and Basdogan, 1997; Kuroda et al., 2013).

Notably, hand exoskeleton is a popular form of the hand interface, and this preference is largely supported by their controllability of each finger joint. It has been designed in various forms depending on its usage, such as teleoperation (master-slave), assistive, and rehabilitation, including VR haptic. As for the hand assistive exoskeleton, it helps patients' fingers to interact with real objects while patients stay passive. Relevant studies are the cable-driven glove type, such as In and Cho (2009) and In et al. (2011), which are actuated by the electric actuators. Whereas, Noritsugu et al. (2004), Kadowaki et al. (2011), and Toya et al. (2011) is the glove type equipped with the pneumatic actuators. The hand rehabilitation exoskeleton allows users to carry out repetitive therapy tasks to treat physical or neurological disabilities. Jones et al. (2010), Chiri et al. (2011), and Li et al. (2011) are the examples of the cable type rehabilitation exoskeleton with the electric actuators. Lastly, Blake and Gurocak (2009), Ben-Tzvi and Ma (2014), In et al. (2015), Jo and Bae (2015), and Hinchet et al. (2018) were proposed as a glove based haptic exoskeleton. Furthermore, several haptic gloves are commercially available, such as Dexmo (Dexta Robotics, Shenzhen, China), HaptX (Seattle, US), and SenseGlove (Senseglove, Delft, Netherlands). Although there is no doubt in the importance of portability and lightness (Kuroda et al., 2013; Sarac et al., 2019), numerous exoskeletal haptic interfaces have overlooked these factors. Nevertheless, this trend is somewhat inevitable, considering the weight and the complexity of the mechanical link structures required to work.

Previous studies on human sensation/perception of fingertip force provide information on how to render haptic feedback to a user's hand with a haptic display. Matsui et al. (2013) showed that both cutaneous and kinesthetic feedback contribute to force perception at the fingertip. Similarly, recent studies demonstrated that the addition of cutaneous feedback to the kinesthetic feedback significantly affects the perception of a static or dynamic virtual object (van Beek et al., 2019; Park et al., 2020). Thus, one has to consider incorporating both cutaneous and kinesthetic feedback to a hand haptic interface. Another crucial but yet thoroughly investigated subject regarding hand haptic interface is where and how to provide the kinesthetic feedback to a fingertip in an optimal manner. Previous physiological studies show evidence that muscle spindles and Golgi tendon organs are responsible for the perception of force or heaviness (Ferrell et al., 1987; Clark et al., 1989; Proske and Gandevia, 2012). When it comes to the haptic interface design, this implies that essentially, kinesthetic feedback has to be provided to multiple joint locations of a finger for an increased proprioceptive acuity. However, it is yet hard to find the reference of providing kinesthetic feedback to multiple joint locations and how to optimize the input.

From the aforementioned studies, we noticed several limitations of the exoskeletal haptic interface: concentration on kinesthetic feedback, complexity and the weight of the system, and the lack of the multimodal rendering method. In the present study, we propose a haptic glove that can provide both cutaneous and kinesthetic feedback to two fingers—a thumb and an index finger. The cutaneous feedback is provided with a contact plate to render contact with a virtual or remote object. The kinesthetic feedback is rendered to PIP and DIP joints. We adopted a tendon-driven mechanism to minimize the weight of the system and to increase the wearability. By using the proposed haptic interface, we introduce a perception-based kinesthetic feedback optimization strategy. While our proposed haptic interface can render kinesthetic feedback to multiple finger joints, there is no previous reference on how to distribute the target force for the target joints for haptic applications. Based on the optimal sensory integration model (Ernst and Banks, 2002), we distribute the target force for the two joints based on the individual force perception data for PIP and MCP joints. In addition to the kinesthetic feedback, we expect that additional cutaneous feedback on a fingertip affects human sensitivity. We evaluate the validity of our proposed method by conducting a set of psychophysics experiments: (1) Mapping the perceived force at the fingertip to the force applied to joints, (2) Perception of force rendered at a single joint (PIP/MCP), (3) Perception of kinesthetic feedback (PIP + MCP), and kinesthetic feedback (PIP + MCP) + cutaneous feedback. The goals of this study are thus, (1) to propose *a wearable multi-modal haptic glove that can provide both cutaneous and kinesthetic feedback*, and (2) to evaluate *the validity of multi-modal haptic feedback approach*.

The rest of this paper is organized as follows. In the next section, we describe the haptic interface in the aspect of hardware setup. Then, we explain haptic rendering strategies, including the perception-based kinesthetic feedback method. Next, an experiment and its results to evaluate our approach are presented. Finally, we conclude our paper with future work.

## 2. Methods

### 2.1. Device Design

**Figure 1:**
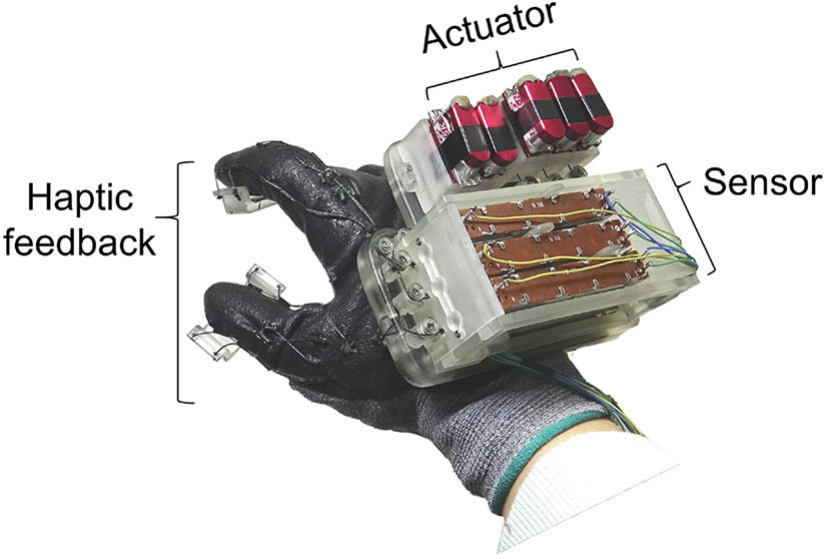
A prototype of the tendon-driven haptic glove. The system consists of three parts: sensor, actuator, haptic feedback.

**Figure 2:**
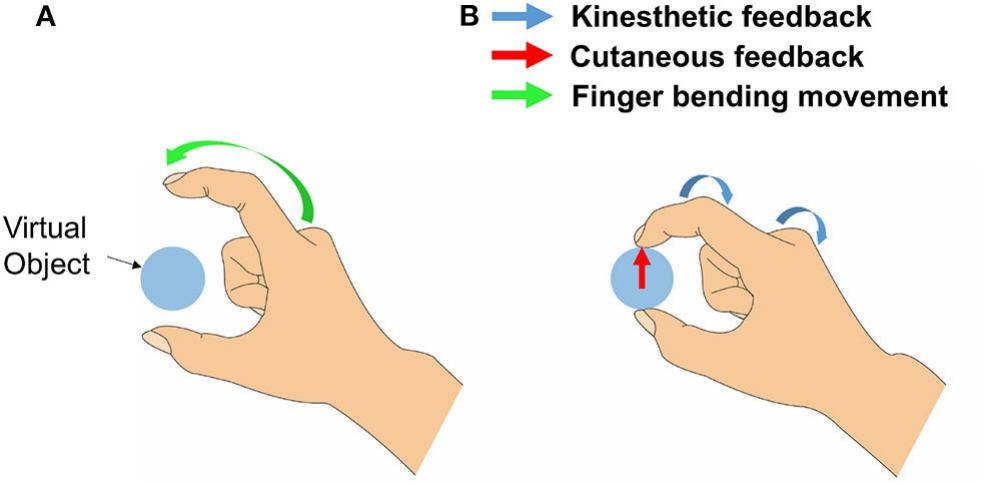
The example of an index finger movement when both cutaneous and kinesthetic feedback is rendered. In **(A)**, a green arrow represents finger movement when a user bends his/her index finger. By the resistive force, the system emulates the sensation of touching a virtual object. Also in **(B)**, blue arrows show kinesthetic feedback rendered on PIP and MCP joints, and a red arrow shows cutaneous feedback rendered on the fingertip.

In the present study, we introduce a haptic interface that renders kinesthetic feedback and cutaneous feedback using a tendon-driven compliant mechanism that can render the normal contact force between the fingertip and a virtual/remote object (Figure 1). The interface was designed based on the human hand skeletal model, where the index finger consists of three phalangeal joints (DIP, MCP, PIP), and thumb consists of two phalangeal joints (DIP, MCP). In our system kinesthetic feedback is rendered on PIP and MCP joints of an index finger, and DIP of a thumb. Also, cutaneous feedback is imparted on distal phalanx of both index finger and thumb. Figure 2 is an example of haptic feedback rendered on an index finger. Both kinesthetic and cutaneous feedback are imparted, and extend the finger by applying the force on finger joints and a fingertip. Here, the “force” refers to resistive force which acts in the direction of constipating the user's finger movement when grasping the virtual/remote object. Furthermore, we aim to impart kinesthetic feedback on multiple joints with optimized torque control by their weight gained from JND.

**Figure 3:**
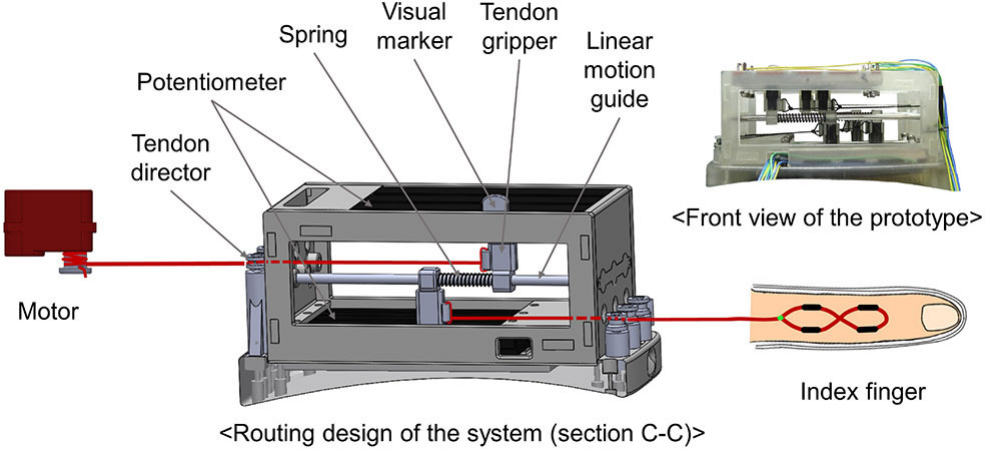
A figure shows a routing design of the system when seen from cross-section (C-C) of Figure 6.

**Figure 4:**
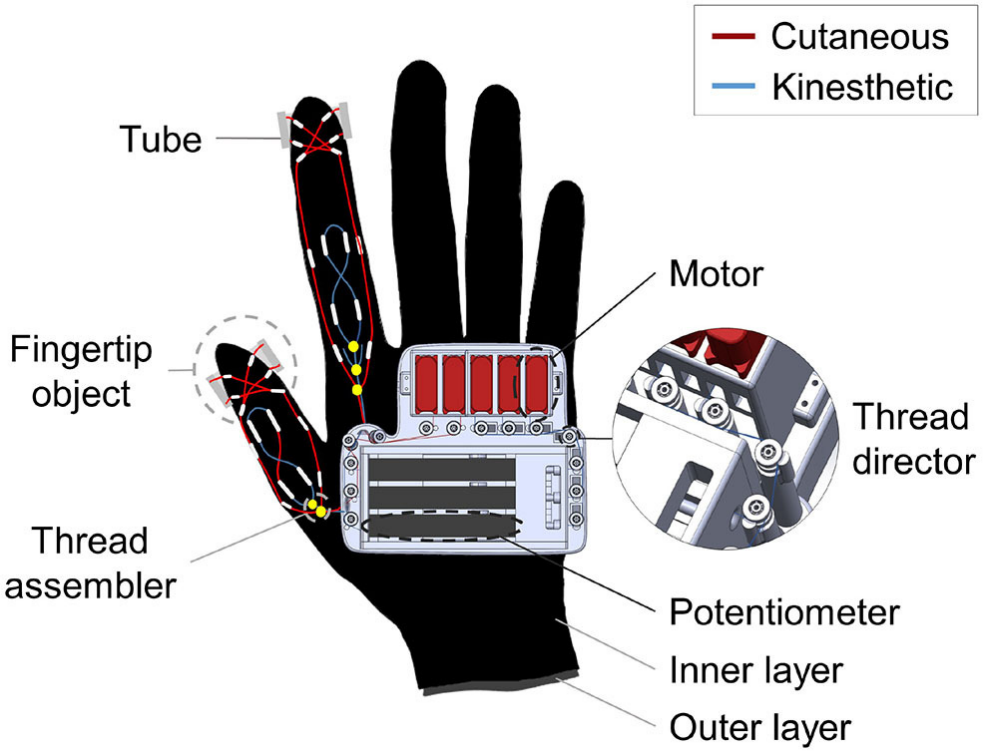
A figure shows an overall specified CAD design of the tendon-driven haptic glove. The system has a bi-layered structure to render independent kinesthetic feedback on PIP and MCP of the index finger. Also, a close-up view of the tendon director is shown in the side. The tendon director has pulleys on top which consists of bearing in order to lessen the friction.

Figure 1 shows a prototype of a tendon-driven haptic interface worn on the dorsal of hands. The module was fabricated with the FDM method (ProJet 3500, 3D SYSTEMS, USA), using VisiJet M3 Crystal as part material. The weight of the system is 230.1g and has a dimension of 101.26 mm × 87 mm (including the glove: 200 mm) × 62.4 mm. For the durability and flexibility of the tendon, we utilized a fishing gut made of polyethylene fibers (TP300M, Seaknight, China). The system consists of three parts: actuator, sensor, and haptic rendering part. As shown in Figure 3, routing of the system starts from the motor where the tendon is winded. The tendon continues to the upper tendon gripper, which moves according to the motor's operation. The other tendon starts from the lower tendon gripper and is connected to the finger joints. The lower tendon gripper moves in accordance with the finger movements. Here, based on the strength obtained from the sensors, actuators pull the tendon and render kinesthetic feedback on fingers. Additionally, cutaneous feedback is imparted on both fingers, while motors are controlled by force-sensing resistors (FSR-402, interlink, Korea) stuck on fingertip object (Figure 4).

**Figure 5:**
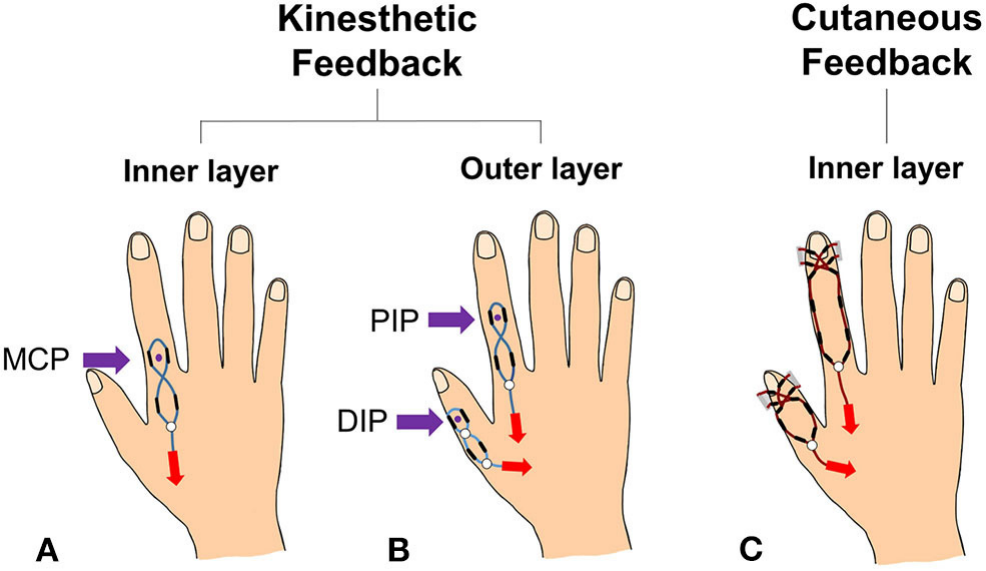
A figure shows a classification of the haptic feedback part to show the bi-layered structure of the proposed haptic interface. **(A)** Is the kinesthetic feedback system of the glove's inner layer, and **(B)** is the kinesthetic feedback system of the outer layer. Also, **(C)** is the cutaneous feedback system on the inner layer.

For the friction loss prevention, there exist tendon directors for angling the tendon, which contain pulley (681-H, NSK, Japan) on top (Figure 4). Furthermore, the glove has a bi-layered (inner layer, outer layer) structure, where each layer is completely separated from each other. The routing system of the PIP joint exists on the outer layer, whereas the system of the MCP joint exists on the inner layer (Figure 5). This structure is devised to avoid interference that can affect each other when rendering kinesthetic feedback on multiple joints of the index finger.

**Figure 6:**
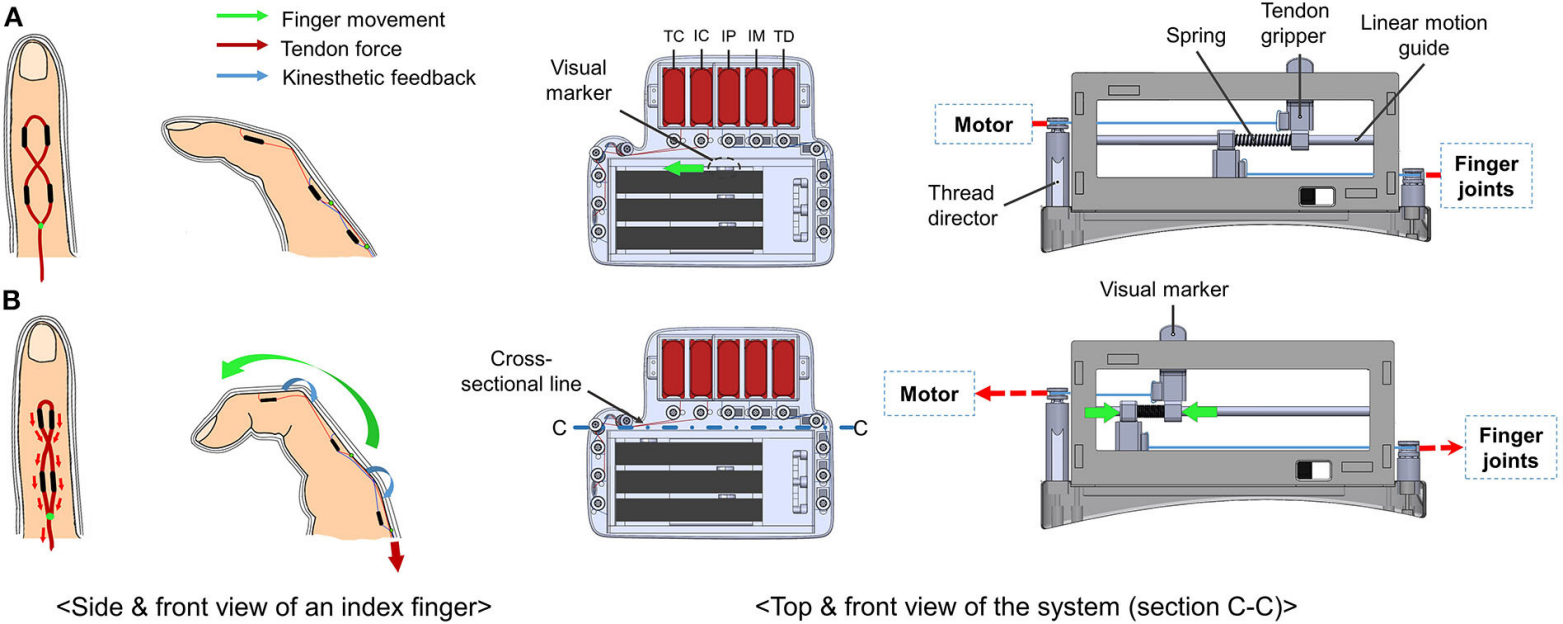
A figure shows the finger movements along with the top view and the cross-sectional diagram (C-C) of the system operation before-**(A)** and after-**(B)** the kinesthetic feedback is rendered on the PIP joint. When the tendon is pulled, and the user's finger bends, the upper and the lower tendon gripper are pulled in the opposite direction to each other. Then, potentiometers sense the tension by their position difference and the coefficient of the compressed spring. Along with this movement, the visual marker shows the movement of the system.

The Actuator part is remotely located from finger joints, which benefits the freedom of motion and lessen the fatigue that can happen due to the weight and volume of them (Figure 4). Also it has five DC motors (hv75K-n, Hitec, Korea) fixed to the motor holders. Among them, two on the left (TC: thumb cutaneous, IC: index finger cutaneous) are used for the cutaneous feedback, and the rest of three (IP: index finger PIP, IM: index finger MCP, TD: thumb DIP) is for the kinesthetic feedback (Figure 6A).

In the case of kinesthetic feedback, sensor part consists of three pairs of potentiometers (PTA4543-2010CIB102, BOURNS, USA), which is bolted to the lower and the upper part of the sensor part (Figure 3). The tendon grippers clench tightly to each potentiometer, and the linear motion guide penetrates them. The upper gripper is connected to motors, and the lower gripper is connected to the fingertip joints. The tendon is tied to each tendon grippers and finished with super glue (Loctite 401, Henkel, Germany). Between these grippers, there exists a spring (WT4-20, MISUMI, Japan). Whenever a user bends his/her fingers, motor winds the tendon, which compresses the spring (Figure 6). By the extent of the compression, it senses the tension of the tendon. Also, we designed a visual marker of the upper tendon gripper to visually show the sensor movements. For controlling the cutaneous feedback, there are FSR sensors for each fingertip (Figure 4). By the magnitude of the pressure between the fingertip and the fingertip object, the actuators are controlled.

Lastly, several polyolefin tubes (KUHS-225G, Unitube, Korea) act as a passage of the tendons for haptic feedback, and they are attached to the glove with super glue. The routing design of the system not only determines the strength, but also an action point of the haptic feedback. Accordingly, the tubes are arranged in different ways depending on the type of haptic feedback (Figure 5). In the case of kinesthetic feedback, a pair of tubes are on DIP, MCP, and a metacarpal bone of the index finger while a pair of tubes are stuck to DIP and metacarpal bones of the thumb. In order to effectively connect the tendons, we used tendon assembler as shown in Figure 4. The tendon is tied to the tendon assembler and passes through each tube, forming the route like an 8- like shape. This form maximizes the force efficiency on the joint by setting a reference point at a lower phalanx, and acting point at an upper joint (Figure 5). For instance, kinesthetic feedback on the MCP joint has its acting point on the MCP joint and the reference point on metacarpal bones. For the cutaneous feedback, four tubes are on distal phalanx of the fingers to equally transfer the tension to the fingertip object Also, we set the tubes along with both sides of the finger to render independent feedback on fingertips without intervening on kinesthetic feedback. By drawing the tendon, the fingertip object touches the fingertip.

### 2.2. Rendering Haptic Feedback to a Finger for a Contact With Virtual Surface

Our proposed haptic interface can render contact with a virtual/remote surface by providing both cutaneous and kinesthetic feedback to two fingers of a user. For a virtual reality application, an optical sensor (e.g., Microsoft Kinect or OptiTrack Motion Capture System) or a magnetic position sensor (e.g., a Polhemus tracking system) can track the fingertip position. If there is a contact between a fingertip avatar and virtual object surface, the contact force at the fingertip is calculated by using a typical spring model as follows:

(1) $$\begin{array}{l} F_{contact}=\{\begin{array}{lc} K\left(x_{p}-x_{f}\right) & \left(contact\right)\\ 0 & \left(otherwise\right) \end{array} \end{array}$$

where *K*, ***x***_*p*_, and ***x***_*f*_ denote the surface stiffness, the contact position at the virtual proxy, and the contact position vectors in a 3D virtual space at the virtual object, respectively (see Ruspini and Khatib, 2001 and Park et al., 2018 for further details). The position of the virtual proxy vector ***x***_*p*_ is typically calculated as a minimum distance point on the object surface from the fingertip position. A user can control the virtual stiffness K to represent a wide range of object surface properties. When rendering the cutaneous contact force at the fingertip, the FSR is used to control the target force to render the contact force in Equation 1. The contact force for the cutaneous feedback is decided as a fraction of the contact force *F*_*n*_ as follows,

(2) $$\begin{array}{r} F_{c}=\alpha_{c}F_{n}, \end{array}$$

where α_*c*_ is the rate of cutaneous hardness, which is a fraction coefficient to determine the perceived hardness of the virtual surface (Park et al., 2018). In the previous study, the contact force with the cutaneous feedback was rendered as ***F***_*c*_ = *K*_*C*_|***x***_*p*_ − ***x***_*f*_|, where *K*_*C*_ is a programmable variable that defines the perceived hardness with the cutaneous feedback. For the kinesthetic feedback, the target force in Equation (1) needs to be distributed as a fraction for each joint. For the distribution, we use an optimal sensory integration model, where the perception of a certain type of stimulus can be modeled as an optimally integrated sum of different signal sources (Ernst and Banks, 2002). When one presses over an object surface with a fingertip, s/he perceives the contact force with the kinesthetic information at finger joints, as well as the cutaneous information at the fingertip. Then, the optimal sensory integration model computes the fractional contribution of the perception at each sensory organ as *w*_*i*_ (0 ≤ *w*_*i*_ ≤ 1) as follows:

(3) $$\begin{array}{c} w_{i}=\frac{1/\sigma_{i}^{2}}{\sum_{i}1/\sigma_{i}^{2}}, \end{array}$$

where σ_*i*_ is the Weber fraction of joint *i*. When we have measurement data of the Weber fraction for multiple fingertip force, we can derive an estimate function of the Weber fraction for a fingertip force *f*_*ft*_ as $\hat{\sigma }\left(f_{ft}\right)$. Then, given a fingertip force *F*_*ft*_, the desired force for a joint *i* is,

(4) $$\begin{array}{r} f_{i}\left(F_{ft}\right)={\hat{\sigma }}_{i}\left(F_{ft}\right)F_{ft}. \end{array}$$

### 2.3. Control of Tension Force and Compensation of Friction

**Figure 7:**
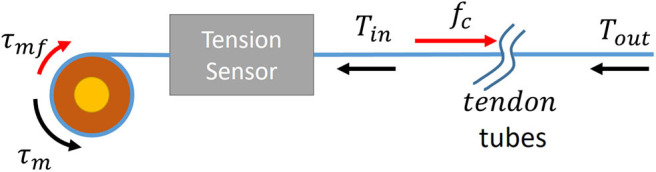
Schematic of forces acting on the tendon-driven haptic system.

This subsection describes how the target force rendered by haptic rendering is exerted to the end-effector with our proposed system. The tendon driven mechanism in the present study entails the issue of friction between the tendon and tubes. Figure 7 shows the schematic of the tendon driven kinesthetic feedback mechanism. At the motor side, the motor torque can be modeled as

(5) $$\begin{array}{r} \tau_{m}=\tau_{mf}+r_{m}T_{in}, \end{array}$$

where τ_*m*_, τ_*mf*_, *r*_*m*_, and *T*_*in*_ indicate the motor torque, Coulomb friction of the motor, the radius of the motor spool, and the tension of the wire between the spool and the tubes, respectively. The force at the end-effector *T*_*out*_ is modeled from the previous studies (Kaneko et al., 1991; Jeong and Cho, 2015), as follows:

(6) $$\begin{array}{r} T_{out}=\left(T_{in}-f_{c}\right)\exp\left(\mu \Phi \right), \end{array}$$

where *f*_*c*_, μ, and Φ are the system friction, friction coefficient, and the wire bending angle.

**Figure 8:**
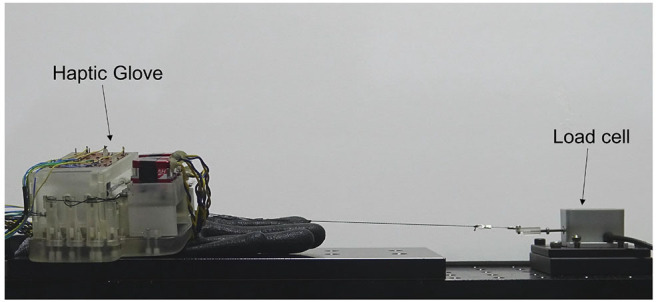
A figure shows an experimental setup for the tension measurement, where a load cell (LTS-2KAZ2, Kyowa, Japan) is connected to our haptic interface.

**Figure 9:**
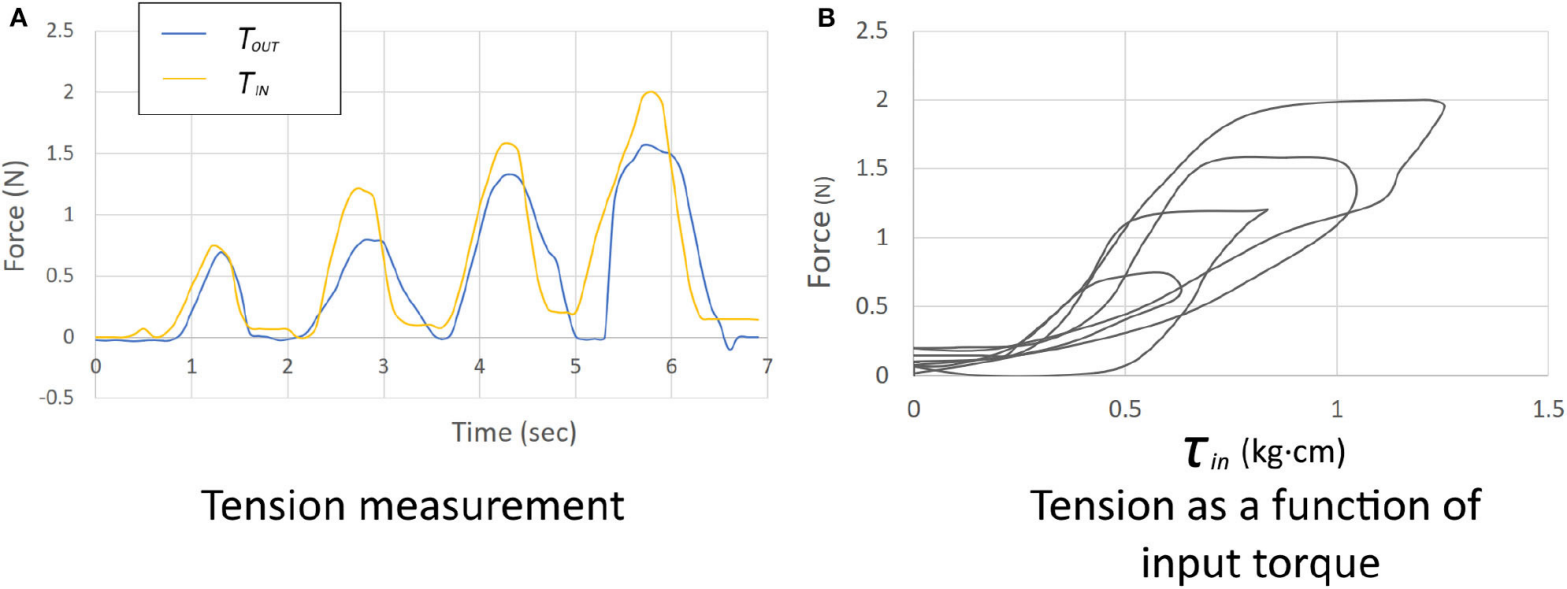
Measured tension sampled at 20 Hz: **(A)** *T*_*in*_ and *T*_*out*_, and **(B)** *T*_*in*_ as a function of τ_*m*_.

To compensate for the system friction *f*_*c*_, we measured *T*_*in*_ and *T*_*out*_ by winding and releasing the tendon (Figure 8). The data was sampled at a frequency of 20 Hz. The tension sensor of the haptic system and a load cell measured *T*_*in*_ and *T*_*out*_, respectively. Figure 9 shows the results of the measurements. Figure 9A shows *T*_*in*_ and *T*_*out*_ as functions of time as the tendon alternately wound and released. Figure 9B shows the relation between the input torque and the tension *T*_*in*_ while winding and releasing the wire. By taking the difference samplings between *T*_*in*_ and *T*_*out*_, we built a linear model to estimate the system friction *f*_*c*_ as follows:

(7) $$\begin{array}{r} {\hat{f}}_{c}=0.2099T_{in}-0.0284. \end{array}$$

Then, given the desired force at the end-effector *f*_*d*_, the desired tension *T*_*in,d*_ is calculated from the Equations (7) and (10), as follows:

(8) $$\begin{array}{r} T_{in,d}=1.267f_{d}\exp\left(-\mu \Phi \right)-0.0359. \end{array}$$

**Figure 10:**
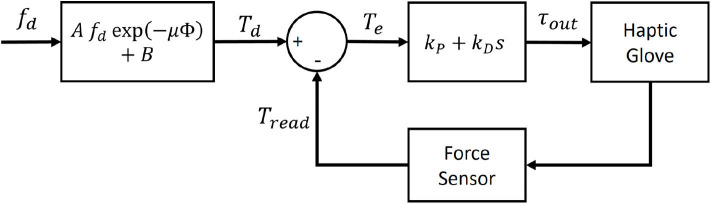
Control block diagram to apply *f*_*d*_ to the target of the haptic glove.

Figure 10 shows the force control block diagram of the haptic system. Given the desired force at the target *f*_*d*_, the target tension *T*_*d*_ is calculated and the motor torque is controlled with a PD controller.

**Figure 11:**
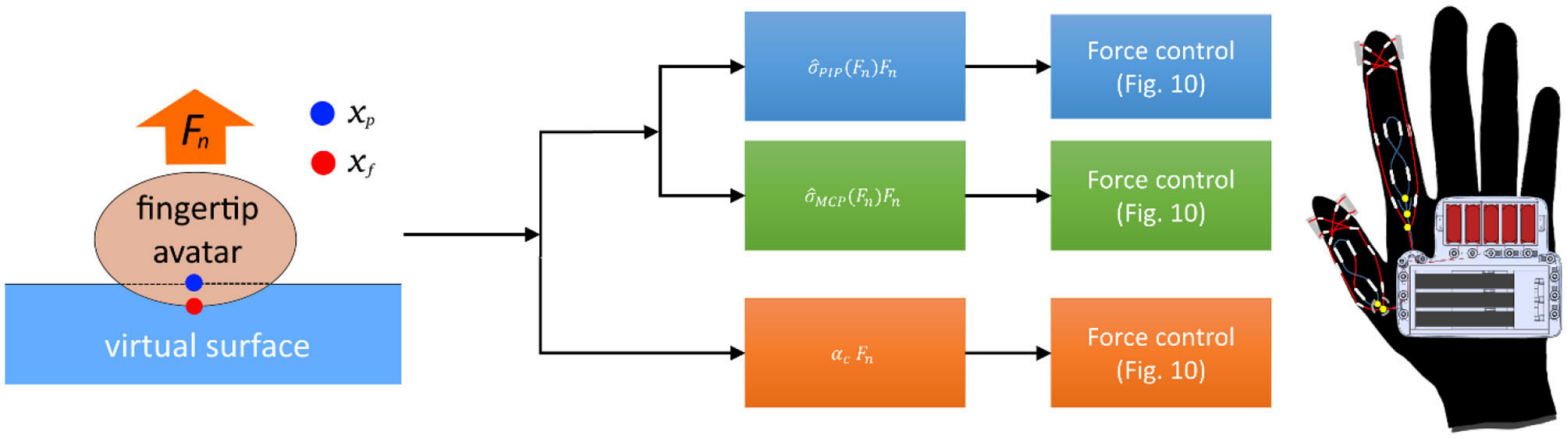
The system architecture for the proposed haptic glove system. The contact force between a user's fingertip avatar and a virtual object is calculated by collision detection (Virtual Environment). Then, the desired force for the cutaneous and kinesthetic feedback is calculated based on the Equations (2) and (4). The calculated force for the target joints and fingertip is then applied to the haptic glove.

Figure 11 shows the overall haptic system architecture, including the virtual environment, controller, and the haptic interface. When a contact is detected by the collision detection, the contact force *F*_*n*_ is calculated by Equation (1). Then, the weight of the kinesthetic feedback for each joint is calculated by Equation (4). Simultaneously, the force for the cutaneous feedback is calculated as a fraction of *F*_*n*_, considering the over-penetration of the fingertip down to the virtual surface. Then, the force controller fed the desired force to the haptic glove.

## 3. Experimental Evaluation of Force Rendering With the Tendon-Driven Haptic Glove

This section describes a series of human haptic perception experiments that evaluated the force rendering with the tendon-driven haptic glove. We first describe the experimental measurement that mapped human perception of input tension applied to PIP and MCP joints, to the reference force applied to the fingertip. Then, we present the experimental results of the force discrimination of human subjects for the two joints. Finally, we evaluate the human perception of the force rendered by our proposed algorithm and haptic glove.

All methods were previously approved by the KIST (Korea Institute of Science and Technology) Institutional Review Board (IRB approval No. 2019-007) and carried out in accordance with the Declaration of Helsinki for research involving human subjects. Informed written consent was obtained from all participants involved in the experiments.

### 3.1. Measurement 1: Mapping the Perceived Force at the Fingertip to the Force Applied to Joints

We use human perception of force at the joints to estimate the contact force at the fingertip as described in the previous section. The experiment was conducted for the index finger. When a set of force is applied to the fingertip, a subject adjusts the force to a specified joint, to be matched to the perceived force at the fingertip.

Total eleven healthy subjects (three females, 21–27 years old) participated in the experiment with informed consent. None of them reported any problem in the sense of touch. We conducted the measurement to achieve a mapping of the kinesthetic feedback at the fingertip to the force exerted to a PIP/MCP joint. The kinesthetic feedback was exerted by a commercially available kinesthetic feedback interface, PHANToM Premium 1.0 (3D Systems Inc., SC, USA). The measurement was conducted for two joints of an index finger. The perceived force at each joint was measured as input torque, for three force values at the fingertip, 0.5, 1.0, and 1.5 N. We used the method of adjustment to let a participant match the perceived force at a fingertip to the one felt at the fingertip (Gescheider, 1985).

**Figure 12:**
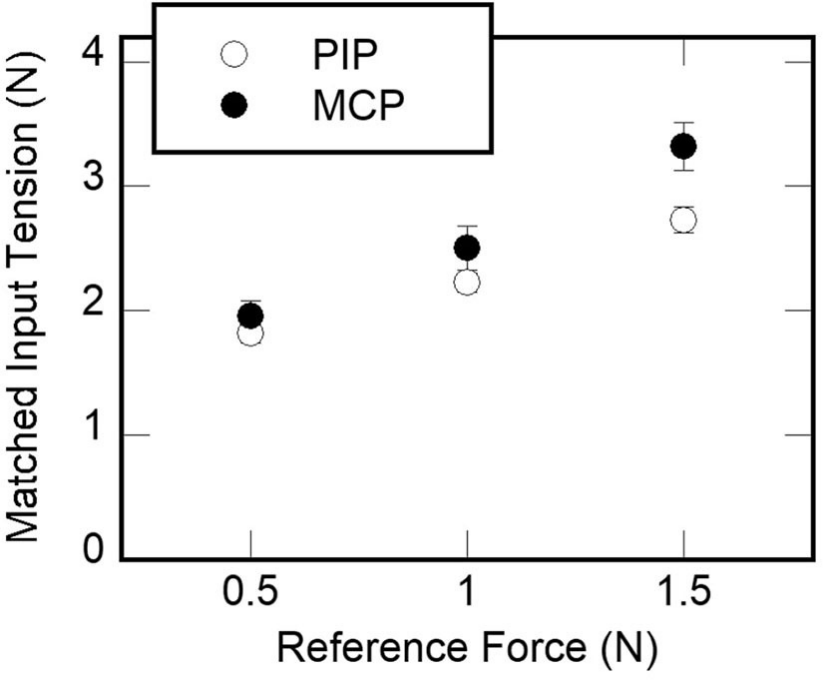
The results of measurements 1. The mean tension force applied to PIP and MCP joints by reference forces applied to the fingertip by a PHANToM force-feedback interface.

Figure 12 shows mean tension force applied to the two joints plotted against the reference force applied to the fingertip. When a two-way repeated measure ANOVA was conducted on the tension force with the factors of the joint and the reference force, both factors had significant effects [*F*_(1, 10)_ = 5.5, *p* = 0.041, η^2^ = 0.36 for the joint; *F*_(2, 20)_ = 61.2, *p* < 0.0001, η^2^ = 0.86 for the reference force]. No significant interaction between the two factors was observed [*F*_(2, 20)_ = 3.45, *p* = 0.052, η^2^ = 0.26]. In a subsequent Bonferroni *post-hoc* test, there was a significant difference in the tension force between the two joints. Also, no reference force pairs were grouped. The result implies that the participants matched the input tension applied to the fingertip to the reference force applied to the two joints in an increasing manner. Also, given the same amount of kinesthetic feedback applied by the PHANToM, the matched tension was smaller for the MCP than the PIP joint.

### 3.2. Measurement 2: Perception of Force Rendered at a Single Joint (PIP/MCP)

This subsection describes an experiment that measured the perception of force applied to a PIP or MCP joint, to derive the relative weight in the Equation (3). The calculated weight of each participants will be applied to force distribution on each finger joints. As with Measurement 1, the experiment was conducted for the index finger.

#### 3.2.1. Experiment Design

We used a standard one-interval two-alternative-forced-choice (1I-2AFC) experimental paradigm or a yes-no experiment to calculated the JND values of force for the two joints. The perception of the joint is characterized as a just noticeable difference (JND), from which we derived the Weber fraction (Macmillan and Creelman, 2004). For the derivation of a JND for a reference, the signal detection theory (SDT) defines the sensitivity index ***d*′**, which is a measure for how well one can discriminate the difference between the reference **α**_**0**_ and a comparison **α**_**0**_** + △α**. The ***d*′** value is calculated from stimulus response matrix, with the hit rate **(*H*)** and the false alarm rate **(*F*)** as follows:

(9) $$\begin{array}{r} {\text{d}}^{\prime }=z\left(H\right)-\;z\left(F\right), \end{array}$$

where ***z*(·)** is the z-score function. Then, the JND is defined as the amount of the stimulus, denoted as (**△α**)_**0**_ increment for ***d*′** = 1. Given the measurement data for a reference and multiple comparison stimuli, the JND value can be estimated as an inverse of the average slope, denoted as $\bar{\delta }$. Weber fraction (**σ**_***s***_) is then estimated as

(10) $$\begin{array}{r} \sigma_{s}=\frac{{\left(△\alpha \right)}_{0}}{\alpha_{0}}. \end{array}$$

assuming the linearity between the *d*′ values and Δα. Then, the relative weight of each finger can be derived from the Equation (3).

#### 3.2.2. Stimuli

The stimuli for the experiment were the force applied to either PIP or MCP joints. The reference force was 1.0 N and the comparison stimuli were 1.2 and 1.5 N (Δα= 0.2 and 0.5 N). Therefore, there were four experiment runs (two joints × two Δα), and each experiment run consisted of 20 experimental trials for the data collection.

#### 3.2.3. Procedure

**Figure 13:**
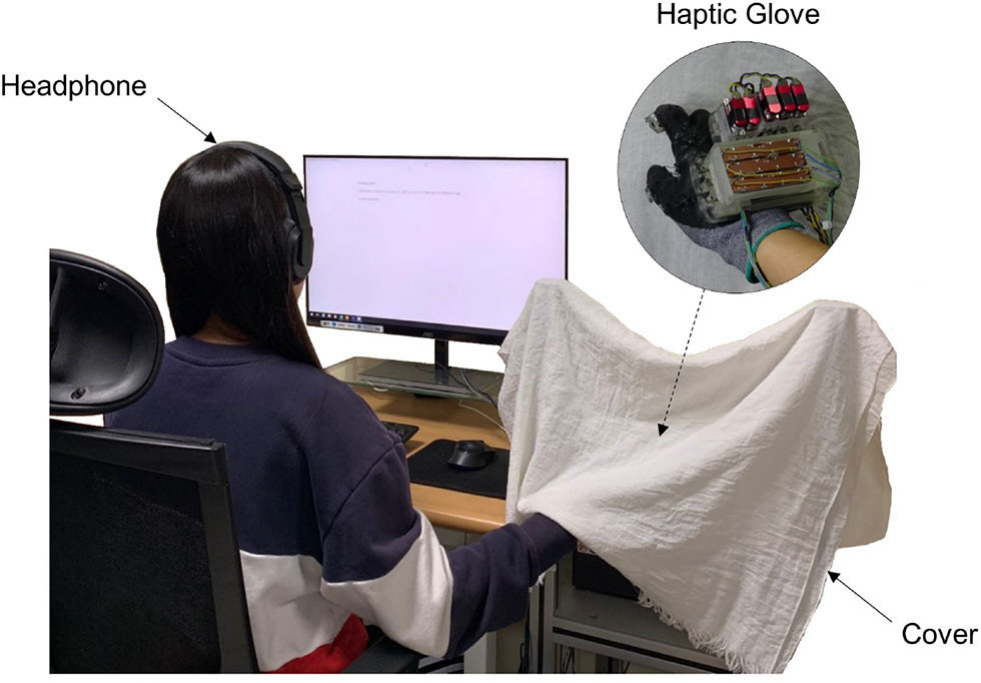
The experimental setup. A participant wears a headphone where white noise is played during the experiment. S/he also wears a haptic glove which is covered in order to block the visual cues.

At the beginning of an experimental run, a participant was seated in front of the experiment computer. S/he put on noise-canceling headphones (MDR10RNC, Sony, Tokyo, Japan), where white noise was played during the experiment to block a possible audio cue from the haptic interface. Then, the participant wore the haptic glove for the experiment (Figure 13).

Prior to the experiment, a training session was available to let the participant be familiarized with the experimental stimuli, the reference, and the comparison stimuli. Throughout the training session, participants were allowed to trigger either reference or comparison stimuli by pressing the “1” (reference) or “2” (comparison) key, respectively, which is in line with the following experimental setting. Also, the caption of the triggered stimulus was shown in the monitor. The participant could feel the stimuli as many times as possible and could move to the main experiment when s/he was ready. On each trial of the experiment, one of the two stimuli (reference/comparison) was presented to the participant, as the participant trigger the stimulus. The stimulus was given randomly with an equal *a priori* probability of 0.5. After having felt the stimulus, s/he was asked to type “1” (reference) or “2” (comparison) key to indicate what the participant felt. The participant can move on to the next phase as soon as they are ready by pressing a spacebar.

#### 3.2.4. Results

**Figure 14:**
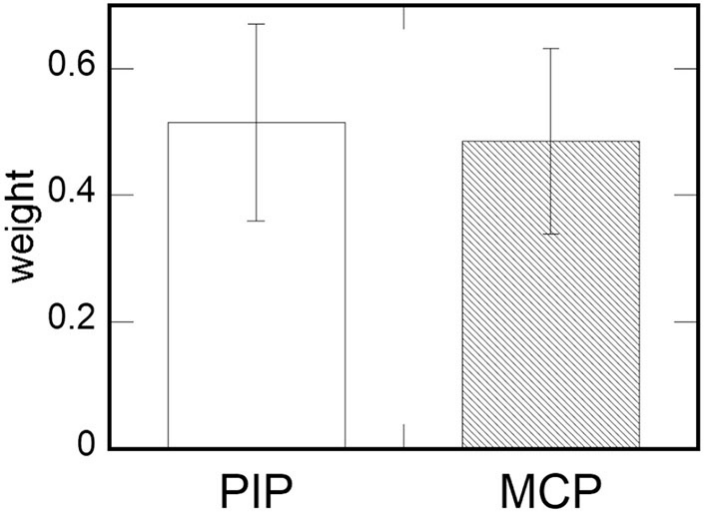
The results of measurements 2. The mean weight of the two joints derived from the force perception data. Error bars indicate standard errors.

From the Weber fraction, we calculated the weight of the force perception for the two joints of each participants (Figure 14) using the equation 3. The result of a paired *t*-test indicates no significant difference in the weight of force perception between the two joints [*t*_(10)_ = 0.23, *p* = 0.83]. The results implies that the participants' ability to perceive the force did not vary significantly by the joint.

### 3.3. Measurement 3: Perception of Kinesthetic Feedback (PIP + MCP), and Kinesthetic Feedback (PIP + MCP) + Cutaneous Feedback

This subsection describes an experiment that evaluated the effect of additional cutaneous feedback to force perception at a fingertip. We experimented with two conditions, (i) kinesthetic feedback (to PIP + MCP joints), and (ii) kinesthetic (to PIP + MCP joints) + cutaneous feedback. Then, we compared the force perception for the two conditions, to evaluate the additional cutaneous feedback.

We evaluated the Weber fraction for the force perception under the two conditions as the perception measure. We used the 1I-2AFC to derive the Weber fraction, meaning that the overall experimental procedure was the same as measurement 2. As with measurements 1 and 2, the experiment was conducted for the index finger.

The participants differed from the measurements 1 and 2. A total of eleven healthy subjects (three females, 21–27 years old) participated in the measurement with informed consent, and none of them has reported any problem in the sense of touch.

#### 3.3.1. Stimuli

The kinesthetic feedback for the experiment were the force applied to both PIP and MCP joints by the force distribution, and the cutaneous feedback was imparted on a fingertip. For the rendering of the kinesthetic feedback to the PIP and MCP joints, we used the weights from measurement 2. Given target force *F*_*target*_, the force applied to each joint *F*_*idx*_ (*idx* ∈ {*PIP, MCP*}) was distributed as *F*_*idx*_ = *w*_*idx*_*F*_*target*_. For the rendering of the cutaneous feedback, we used the FSR sensor at the fingertip to control the contact force at the fingertip. We set α_*c*_ in Equation (2) to be 1.0, considering no modulation of the perceived force at the fingertip. The reference force was 0.5 N and the comparison stimuli were 1.0 and 1.5 N (Δα = 0.5 and 1.0 N). Therefore, there were four experiment runs (two conditions × two Δα), and each experiment run consisted of 20 experimental trials for the data collection.

#### 3.3.2. Results

**Figure 15:**
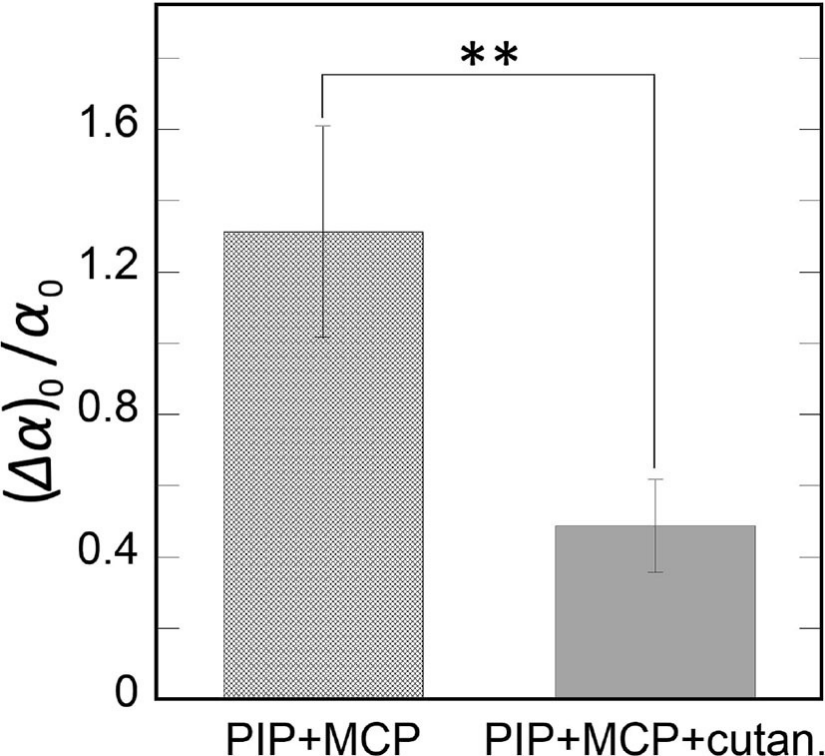
The results of measurement 3. The mean Weber fractions are plotted by the two experimental conditions kinesthetic feedback (PIP + MCP), and kinesthetic + cutaneous feedback (PIP + MCP + cutaneous). Error bars indicate the standard errors. **p < 0.01.

Figure 15 shows the mean Weber fraction of the kinesthetic feedback (PIP + MCP), and kinesthetic feedback + cutaneous feedback (PIP + MCP + cutan.). When we conducted a paired *t*-test, a significant difference in the Weber fraction between the two conditions was found [*t*_(10)_ = 2.839, *p* = 0.008] between the two conditions. The result indicates that additional cutaneous feedback on a fingertip increased the acuity of the human force perception.

### 3.4. Evaluation of Haptic Rendering Method

To evaluate the validity of our proposed haptic system, we conducted subjective rating of kinesthetic feedback rendered in random order with four different methods: (i) PIP joint kinesthetic feedback (PIP), (ii) MCP joint kinesthetic feedback (MCP), (iii) PIP plus MCP joint kinesthetic feedback (PIP + MCP), and (iv) PIP plus MCP kinesthetic feedback and cutaneous feedback (PIP + MCP + cutaneous). The same subjects who took part in the experiment 1, 2 participated in the rendering method evaluation. A participant was allowed to repeat each haptic rendering method with keyboard input and feel virtual surface as much as they wanted. After feeling each feedback, the participant rated each method by answering questions on (1) the realism of the contact force, and (2) representation of the contact force at the fingertip. We used the 5-pt Likert scale for the subjective rating, and the questionnaires were balanced with negative questions. The questionnaires were *Q1: Is the contact force realistic? Q2: Can you feel the contact force at the fingertip? Q3: Is the contact force unrealistic?* (the negative question of Q1), and *Q4: Can you not feel the contact force at the fingertip?* (the negative question of Q2).

**Figure 16:**
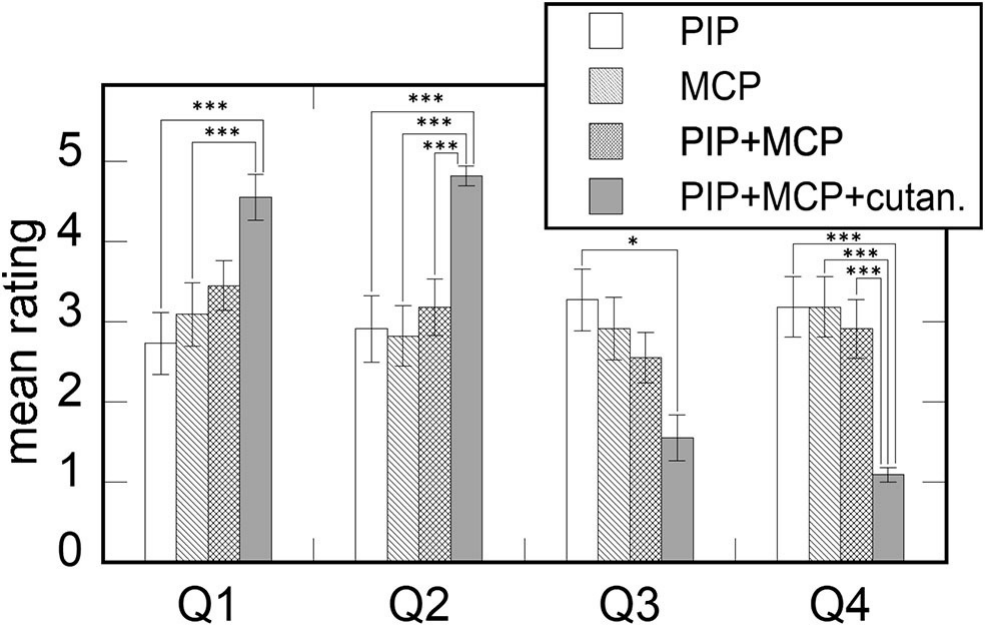
The mean rating of the four haptic feedback methods to render the contact force at the fingertip. The questionnaires are Q1: “Is the contact force realistic?,” Q2: “Can you feel the contact force at the fingertip?,” Q3: “Is the contact force unrealistic?” (the negative question of Q1), and Q4: “Can you not feel the contact force at the fingertip?” (the negative question of Q2). Error bars indicate the standard errors. *p < 0.05, ***p < 0.001.

Figure 16 shows the results of the subjective rating. The result of a one-way repeated measure ANOVA for all questionnaires shows the significant effect of the rendering method [*F*_(3, 30)_ = 9.41, *p* < 0.001, η^2^ = 0.49 for Q1; *F*_(3, 30)_ = 9.66, *p* < 0.001, η^2^ = 0.66 for Q2; *F*_(3, 30)_ = 8.3, *p* < 0.001, η^2^=0.45 for Q3; *F*_(3, 30)_ = 23.21, *p* < 0.001, η^2^ = 0.7 for Q4]. For Q1 and Q3, the result of an additional Bonferroni test indicates that the ratings of PIP + MCP and PIP + MCP + cutaneous are grouped together. For Q2 and Q4, the rating of PIP + MCP + cutaneous was significantly different than those of other rendering methods. Overall, the addition of cutaneous feedback to the kinesthetic feedback led the participants to feel the contact force more realistic than without the feedback. The cutaneous feedback also benefitted the vividness of contact force at the fingertip. Furthermore, with the kinesthetic feedback to the two joints, the participants tended to rate the realism of the contact force higher than the feedback at one joint.

## 4. Concluding Remarks

In this paper, we propose a haptic glove that can provide both force and cutaneous feedback to a user's index finger and thumb using a tendon-driven mechanism. The kinesthetic feedback of the haptic glove is implemented by exerting force to two joints of a finger, PIP and MCP joints. Also, a contact plate located at the fingertip provides the cutaneous feedback by pressing the user's skin by pulling the tendon. We also presented a haptic rendering algorithm to distribute the kinesthetic feedback to two joints optimally. Overall, we proposed haptic rendering system using optimized force distribution for kinesthetic feedback along with cutaneous feedback. Moreover, we evaluated the proposed system and algorithm, and the result indicated the increased realism and perceived acuity of contact force with our proposed system.

Also, the improved realism and the perceived acuity of the contact force with the our haptic glove system can be explained with haptic perception mechanism. Previous studies on the perception of an object's properties indicate that the human CNS often integrates both the sensory information with different modality, especially when they are cutaneous and kinesthetic information (Frisoli et al., 2011; Park et al., 2013, 2018). Our proposed haptic system mechanism emulated the tactile stimuli evoked when one is touching an object with a fingertip. As shown from experiment 3, the additional cutaneous feedback to the kinesthetic feedback for PIP/MCP joints significantly improved the sensitivity of human force perception at the fingertip. Moreover, the participants of our study are thought to have rated the kinesthetic feedback with the highest matching score as more realistic. Similarly, we can explain the grouping of PIP + MCP and PIP + MCP + cutaneous condition for the realism rating by the higher matching of the kinesthetic sensation of touching an object with a fingertip, than the rendering condition for one finger. As a majority of the studies regarding wearable haptic interfaces concentrates on kinesthetic feedback, the result of our study indicates why additional cutaneous feedback to the kinesthetic feedback should not be neglected for the acuity of human perception.

**Figure 17:**
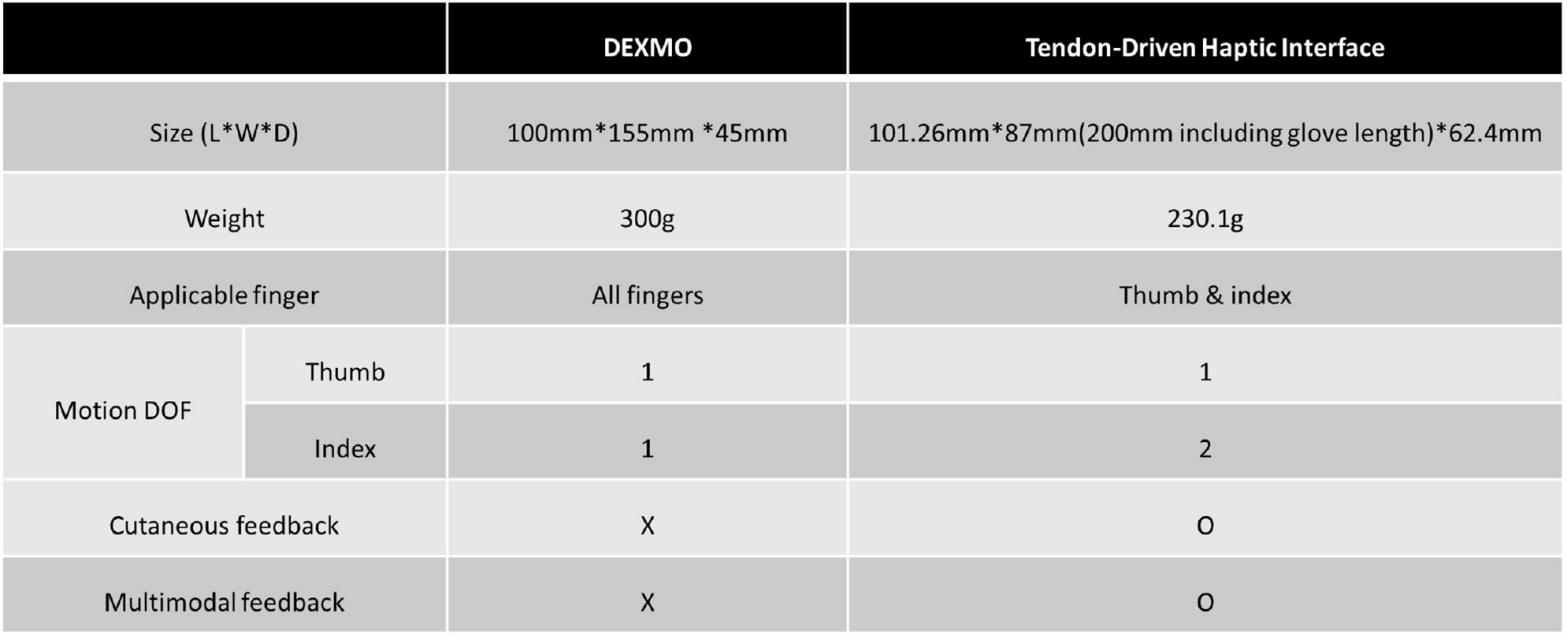
The table shows a comparison of our proposed haptic interface with commercial product Dexmo (Dexta Robotics, Shenzhen, China).

Figure 17 shows a table which is the comparison between our proposed haptic interface and a commercial product, Dexmo. As shown in the table, our proposed interface has a smaller volume and weight than Dexmo. The main advantage of our system is the two motion DOF of the index finger. This multi-modal feedback has the benefit of imparting delicate sensations. Furthermore, the addition of cutaneous feedback can enhance a sense of presence than just applying force feedback. However, Dexmo can be applied to all fingers, whereas our system can only be used on the index finger and thumb. Therefore, our future work will be focusing on devising the system applicable on five fingers.

We are planning to investigate further the perception of the tactile stimuli for a wearable haptic glove system. We will continue to study the human perception of force exerted to multiple locations for a wider range of reference force. Then, a more general force discrimination data will become available, leading to the derivation of the optimal force distribution law. Then, our future work will rigorously evaluate the validity of the perception-based kinesthetic feedback optimization strategy. Furthermore, the mechanism of the haptic glove will be improved by using a soft and compact sensor for the force, such as a piezoelectric force sensor.

## Data Availability Statement

The datasets generated for this study are available on request to the corresponding author.

## Ethics Statement

The studies involving human participants were reviewed and approved by KIST (Korea Institute of Science and Technology) Institutional Review Board. The patients/participants provided their written informed consent to participate in this study.

## Author Contributions

SB and JP conceived the research idea and prepared for the experiment. SB, SP, and JP setup the system concept. SB implemented the hardware. JP analyzed the results. All authors reviewed the manuscript.

## Conflict of Interest

The authors declare that the research was conducted in the absence of any commercial or financial relationships that could be construed as a potential conflict of interest.
