# Corrigendum: Haptic Glove Using Tendon-Driven Soft Robotic Mechanism

**DOI:** 10.3389/fbioe.2020.630005

**Published:** 2020-12-11

**Authors:** Siyeon Baik, Shinsuk Park, Jaeyoung Park

**Affiliations:** ^1^Robotics and Media Institute, Korea Institute of Science and Technology, Seoul, South Korea; ^2^Department of Mechanical Engineering, Korea University, Seoul, South Korea

**Keywords:** haptic interface, tendon-driven mechanism, wearable interface, cutaneous feedback, kinesthetic feedback

In the original article, there was a mistake in the legend for Figure 16 as published. We made an error on the numeric value of the significance level. The correct legend appears below.

“The mean rating of the four haptic feedback methods to render the contact force at the fingertip. The questionnaires are Q1: “Is the contact force realistic?,” Q2: “Can you feel the contact force at the fingertip?,” Q3: “Is the contact force unrealistic?” (the negative question of Q1), and Q4: “Can you not feel the contact force at the fingertip?” (the negative question of Q2). Error bars indicate the standard errors. ^*^p < 0.05, ^***^p < 0.001.”

In the original article, the wrong version of Figure 11 was published. The correct Figure 11 appears below.

**Figure 11:**
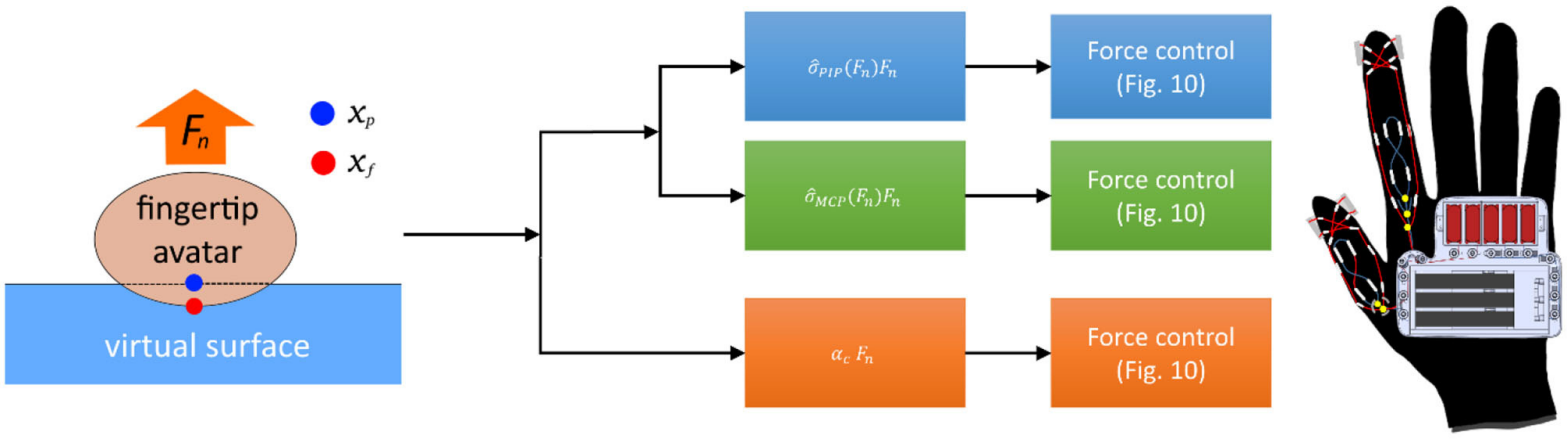
The system architecture for the proposed haptic glove system. The contact force between a user's fingertip avatar and a virtual object is calculated by collision detection (Virtual Environment). Then, the desired force for the cutaneous and kinesthetic feedback is calculated based on the Equations (2) and (4). The calculated force for the target joints and fingertip is then applied to the haptic glove.

In the original article, there was an error. We made an error in the nomenclature of the variable. A correction has been made to **3. Experimental Evaluation of Force Rendering With the Tendon-Driven Haptic Glove, 3.2 Measurement 2: Perception of Force Rendered at a Single Joint (PIP/MCP)**, **3.2.1 Experiment Design**, paragraph 1:

“We used a standard one-interval two-alternative-forced-choice (1I-2AFC) experimental paradigm or a yes-no experiment to calculated the JND values of force for the two joints. The perception of the joint is characterized as a just noticeable difference (JND), from which we derived the Weber fraction (Macmillan and Creelman, 2004). For the derivation of a JND for a reference, the signal detection theory (SDT) defines the sensitivity index ***d*′**, which is a measure for how well one can discriminate the difference between the reference **α**_**0**_ and a comparison **α**_**0**_**+△α**. The ***d*′** value is calculated from stimulus response matrix, with the hit rate **(*H*)** and the false alarm rate **(*F*)** as follows:

(9) $$\begin{array}{r} {\text{d}}^{\prime }=z\left(H\right)-\;z\left(F\right), \end{array}$$

where ***z*(·)** is the z-score function. Then, the JND is defined as the amount of the stimulus, denoted as (**△α**)_**0**_ increment for ***d*′** = 1. Given the measurement data for a reference and multiple comparison stimuli, the JND value can be estimated as an inverse of the average slope, denoted as $\bar{\delta }$. Weber fraction (**σ**_***s***_) is then estimated as

(10) $$\begin{array}{r} \sigma_{s}=\frac{{\left(△\alpha \right)}_{0}}{\alpha_{0}}. \end{array}$$

assuming the linearity between the *d*′ values and Δα. Then, the relative weight of each finger can be derived from the Equation (3).”

The authors apologize for these errors and state that this does not change the scientific conclusions of the article in any way. The original article has been updated.

